# Supplementary material for: Exercise Fat Oxidation Is Positively Associated with Body Fatness in Men with Obesity: Defying the Metabolic Flexibility Paradigm
Source: Int J Environ Res Public Health. 2021 Jun 29;18(13):6945. doi: 10.3390/ijerph18136945 (PMC8297250; doi:10.3390/ijerph18136945)
Supplement: Supplementary file 1 [file ijerph-18-06945-s001.zip › Figure S1.pdf]

Supplementary File S3

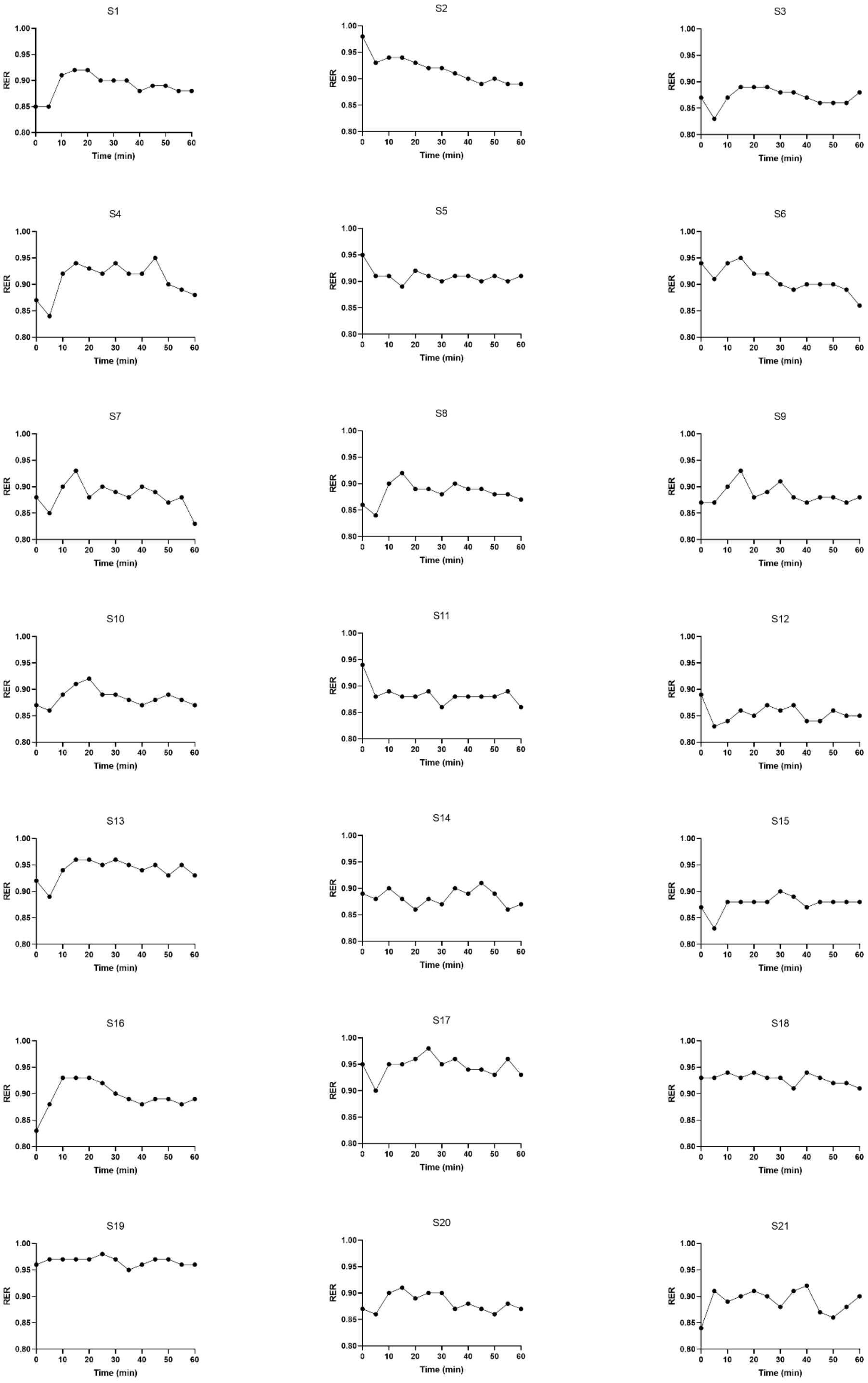

Figure S1. Participants respiratory exchange ratio kinetics during the 60 min FATmax trial.
