# Supplementary material for: Exercise Fat Oxidation Is Positively Associated with Body Fatness in Men with Obesity: Defying the Metabolic Flexibility Paradigm
Source: Int J Environ Res Public Health. 2021 Jun 29;18(13):6945. doi: 10.3390/ijerph18136945 (PMC8297250; doi:10.3390/ijerph18136945)
Supplement: Supplementary file 1 [file ijerph-18-06945-s001.zip › Supplementary File S1.pdf]

CUESTIONARIO DE ACTIVIDAD FÍSICA (CAF)

Nombre: ___________________________ Fecha y día de la semana: _____________________

Instrucciones: Cada columna de la izquierda corresponde a una hora determinada del día. Cada celda de la derecha corresponde a un periodo de 15 minutos de cada hora. Escriba en cada celda vacía la actividad física realizada para cada periodo de 15 minutos. Si la actividad registrada en la subsiguiente celda es la misma trace solo una línea hasta que cambié de actividad.

Hora	Periodo de cada hora en minutos			
0	0-15	16-30	31-45	46-60
1				
2				
3				
4				
5				
6				
7				
8				
9				
10				
11				
12				
13				
14				
15				
16				
17				
18				
19				
20				
21				
22				
23				
