# Supplementary material for: Exercise Fat Oxidation Is Positively Associated with Body Fatness in Men with Obesity: Defying the Metabolic Flexibility Paradigm
Source: Int J Environ Res Public Health. 2021 Jun 29;18(13):6945. doi: 10.3390/ijerph18136945 (PMC8297250; doi:10.3390/ijerph18136945)
Supplement: Supplementary file 1 [file ijerph-18-06945-s001.zip › Supplementary File S2.pdf]

CUESTIONARIO DE SALUD

Historia Médica e informe de Lesiones

Esta forma debe de ser completada por el padre o tutor antes de visitar al Médico General

Fecha______________________________________

Nombre completo______________________________________ Sexo: Hombre Mujer

Fecha de nacimiento _____________________________ Lugar de nacimiento _____________________________

DD MM AA

Dirección:

Calle y Número____

Código Postal __________________ Colonia ___________________________

Ciudad __________________________ Estado __________________________

Teléfono particular ( ) ___________________

Teléfono de trabajo ( )____________________ ext.______________

Teléfono celular ________________________

Servicio Médico con el que cuenta:

IMSS ISSSTE Pensiones Otro _______

Número o clave del servicio médico: ____

Fecha del último examen médico ____ ____ ____

DD MM AA

Fecha del último examen dental ____ ____ ____

DD MM AA

Alergias: ____

Medicamentos que está tomando actualmente:

____

En caso de urgencia notificar a:	
Nombre	
Dirección	
Teléfono (s)	
Parentesco	

Historia familiar

Por favor, identifique cualquier problema de salud que haya ocurrido en su familia (padres, abuelos ó hermanos consanguíneos.							
			quién				quién
Alguien menor a 50 años ha muerto súbitamente	si	no		Alergias o asmas	si	no	
Alta presión arterial	si	no		Anemias	si	no	
Problemas de corazón	si	no		Diabetes	si	no	
Cáncer o tumores	si	no		Epilepsia	si	no	
Migrañas	si	no		Desordenes de riñón y vejiga	si	no	
Problemas emocionales	si	no		Desórdenes de estómago	si	no	
				Alteraciones genéticas	si	no	
Número de hermanos y hermanas							

Historia personal

Ha tenido o tiene alguno de los siguientes problemas de salud		
si	no	Dificultades con sus ojos o con la visión
si	no	Dificultades con su nariz o garganta
si	no	Problemas con sus oídos
si	no	Dolor de cabeza, mareos, debilidad, fatiga o problemas de coordinación o equilibrio (subraye cuáles)
si	no	Adormecimiento en cualquier parte de su cuerpo
si	no	Peligro de conmoción (pérdida de conocimiento) ó algo de temblor de alguna parte de su cuerpo
si	no	Tos, dificultad de respirar, dolor en el pecho o palpitaciones
si	no	Poco apetito, vómito, dolor abdominal, estreñimiento (subraye)
si	no	Rigidez muscular, inchamiento, dolor de músculos y /o huesos (subraye cuáles)
si	no	Algún problema de la piel como dolor, comezón, enrojecimiento o sensación de calor, etc.
si	no	¿Algún otro síntoma?

Ha tenido ó le han dicho que ha tenido o consultado con un médico por alguno de los siguientes padecimientos:		
si	no	Diabetes, bocio (hipo o hipertiroidismo) o alguna enfermedad de alguna glándula endocrina
si	no	Epilepsia
si	no	Desordenes nerviosos o cualquier enfermedad del cerebro o sistema nervioso
si	no	Problemas del corazón o fiebre reumática
si	no	Venas varicosas, flebitis o hemorroides
si	no	Alguna enfermedad de la sangre, formación fácil de moretones o tendencia de sangrado
si	no	Tuberculosis, asma o cualquier enfermedad de los pulmones o alteraciones del Sist. Respiratorio

si	no	Úlceras o cualquier enfermedad del estómago, intestino hígado o vesícula biliar
si	no	Orina con azúcar, sangre o albúmina ó cualquier enfermedad de los riñones u órganos genitourinario
si	no	Artritis, reumatismo, o algún lesión o enfermedad en los huesos, articulaciones, espina dorsal o espalda
si	no	Hernia o cualquier enfermedad de los músculos o la piel
si	no	Cáncer, tumor o cualquier crecimiento en el cuerpo
si	no	Alguna lesión en la cabeza que le ha ocasionado un mareo severo, pérdida de la memoria, vómito, pérdida de la conciencia o requerido atención médica u hospitalización.
si	no	Problemas con deshidratación (pérdida excesiva de agua y/o sales).
si	no	Alguna vez ha tenido problemas de regulación de su temperatura (incremento súbito y sin causa aparente de la temperatura de su cuerpo arriba de 40.5 °C.
		En caso de que si ¿fue hospitalizado?
si	no	Algún otro desorden de control de la temperatura. Especifique
si	no	Ha estado hospitalizado para ser observado o recibir tratamiento de alguna enfermedad
si	no	¿Ha cambiado su peso en el último año? Perdido_______kg. Ganado ________ kg.
si	no	¿Qué explicación le da a este cambio de peso? ____
si	no	¿Está últimamente más sediento que anteriormente?
si	no	¿Estas actualmente en un programa de pérdida de peso?
		Si tu respuesta es si. ¿Cuál es tu esquema? Dieta____ Ejercicio______ Ambos______

Drogas, complementos alimenticios y agentes varios.		
si	no	¿Está tomando algún medicamento?
si	no	¿Está tomando alguna vitamina?
si	no	¿Esta tomando algún estimulante (anfetamina, modafinilo, cocaína, LSD, otros)?
si	no	¿Esta tomando algún agente anabólico (medicamento o suplemento estimulador del crecimiento)
si	no	¿Está tomando pastillas para dormir?
si	no	¿Está tomando algún otro medicamento que se lo haya indicado un médico? ¿Cuál(es)?
si	no	¿Está tomando algún medicamento no indicado por ningún médico? ¿Cuál(es)?
si	no	¿Fuma?
si	no	¿Toma? Si su respuesta es sí ¿Cuánto por semana?
si	no	Alguna vez algún médico le ha dicho que no practique algún(os) deporte(s) por algún período de tiempo. Si su respuesta es si.<br>¿Cuál(es) deporte(s)?

		¿Cuánto tiempo?
si	no	¿Usa lentes de contacto cuando practica deporte?

Datos Ginecológicos		
		A qué edad empezó a menstruar
		A que edad sus períodos fueron regulares
si	no	Tiene dolores o calambres durante tus períodos menstruales
si	no	Presenta anormalidades menstruales, p Ej. sangrados muy grandes
si	no	Tiene algún flujo o secreción vaginal
si	no	Está tomando pastillas anticonceptivas
si	no	Tiene alguna protuberancia o dolor en alguno o ambos senos
si	no	¿Ha estado embarazada? ¿Cuántos hijos tiene?
si	no	Se encuentra en su período de menopausia o postmenopáusico
si	no	Presenta problemas físicos o emocionales por está causa
si	no	Esta tomando algún medicamento por esta causa. ¿Cuáles?
		Tiene algún otro problema ginecológico. Indique cuál
		Indique la última fecha de su examen papanicolao

Traumas y lesiones corporales		
si	No	¿Se ha lesionado, alguna vez, sus hombros, brazos, codos, o muñecas?
		¿Si su respuesta es si, la lesión lo incapacito por 1 semana o más?
si	no	¿Se ha lesionado, alguna vez, la cabeza, la columna a nivel cervical, toráxico o lumbar o en la región sacro ilíaca?
si	no	¿Tiene algún dolor en su espalda?
si	no	Si su respuesta es si. ¿Con que frecuencia?<br>a) rara vez b) ocasionalmente c) frecuentemente d) solo después de ejercicio vigoroso.
si	no	¿Se ha lesionado, alguna vez, su cadera, rodilla, tobillo o pie?
		Si su respuesta fue sí, ¿lo incapacitó por una semana o más?
si	no	¿Se ha lesionado, alguna vez, los cartílagos (meniscos) de alguna articulación?
si	no	¿Ha tenido alguna vez problemas con su rótula (condromalacia o dislocación, etc.)?
si	no	¿Le han dicho alguna vez que los ligamentos de sus rodillas se han dislocado?
si	no	¿Le han dicho alguna vez que le truena la rodilla?
si	no	¿Tiene un perno, clavo o placa en su cuerpo como resultado de alguna intervención quirúrgica para unir 2 huesos?
si	no	¿Ha tenido alguna fractura en los últimos 2 años? Si su respuesta es sí, especifica donde y cuando.
si	no	¿Ha sido sometido a alguna intervención quirúrgica? Si su respuesta es si, especifique por qué y cuándo.

Carta Médica: Resumen cronológico de las enfermedades y lesiones contestadas con un SI		
Fecha	Nombre del Médico y dirección que lo atendió (consultorio, hospital, etc)	Naturaleza de la enfermedad o lesión

____

NOMBRE Y FIRMA DEL ATLETA

Traducido y adaptado de: Physiological Testing of the High-Performance Athlete. Published for the Canadian Association of Sports of Science (1991). 2a Ed. McDougall J D,Wenger H A, Green H J. Tr Hernández R P. Ed. Human Kinetics. Champaing IL.
