# Supplementary material for: Exercise Fat Oxidation Is Positively Associated with Body Fatness in Men with Obesity: Defying the Metabolic Flexibility Paradigm
Source: Int J Environ Res Public Health. 2021 Jun 29;18(13):6945. doi: 10.3390/ijerph18136945 (PMC8297250; doi:10.3390/ijerph18136945)
Supplement: Supplementary file 1 [file ijerph-18-06945-s001.zip › Table S1.pdf]

**Supplementary File S4.**

**Table S1.** Bivariate correlations between metabolic flexibility markers

| | **Latency to $RER_{peak}$ (min)** | **Fat Oxidation Increment (ΔRER)** |
|---|---|---|
| MFO (mg·kg $FFM^{-1}$·$min^{-1}$) | −0.04 | 0.13 |
| TFO (mg·kg $FFM^{-1}$·$min^{-1}$) | 0.06 | −0.03 |

Maximal (MFO) and total (TFO) fat oxidation (60 min).
